# Urban Health: A Prac-tical Application for Clinical-Based Learning

**DOI:** 10.5195/jmla.2025.2179

**Published:** 2025-04-18

**Authors:** Endah Fitriasari

**Affiliations:** 1 endahfitriasari1605@gmail.com, Basic and Medical-Surgical Nursing, STIKes Maluku Husada, Indonesia

Cynthera McNeill, Umeika Stephens, and Tara Walker of Wayne State University, and authors of *Urban Health: Practical Applications for Clinical-Based Learning.* Published in 2022 by Wayne State University's Digital Publishing House, this book is freely accessible as an open-access textbook and is intended to educate medical professionals, especially those specializing in urban medical environments. This book aims to fill the gap in knowledge about the challenges of urban healthcare in medical education. The book deals with various interdisciplinary fields relevant to the target population, such as medical education, public health, medical humanities, and community nursing, which once again addresses the complexity of groups that are underestimated in urban healthcare disparities.

Healthcare in urban environments has a distinct set of challenges specific to socioeconomic, environmental, and systemic factors that disproportionately impact relatively underserved populations. Urbanization is a growing trend in the global population and requires a focused approach to health professional education that combines social determinants of health, care disparities, and medical practice in urban environments. With this in mind, they wrote a textbook on learning through clinical-based applications entitled Urban Health. Most health programs cover health disparities in theory, but few have a formal and direct approach to addressing them in a clinical setting. This book aims to meet that need by combining relevant case studies, real-world examples, and interactive learning exercises to help readers translate their knowledge into strategies that can be applied in urban healthcare practice. The book is written in a way that will educate doctors, nurses, and healthcare administrators about the historical, systemic, and policy-driven determinants that affect the health of urban populations.

*Urban Health: Practical Applications for Clinical-Based Learning* highlights the role of cultural competence, patient-provider trust, and interdisciplinary collaboration in relation to better urban health outcomes. The authors combine clinical experience with social science and insights from policy analysis to prepare future health professionals to provide equitable, compassionate, and effective care in urban settings. The book consists of seven chapters, each of which addresses an important component of urban health care.

Chapter 1 introduces the nature of urban health, highlights the differences between urban and rural health care settings and frames the urban health penalty. Terms directly related to urbanity indicate the greater burden of chronic disease, poorer access to medical services, and endemic health inequities observed in urban populations. Using Detroit as a case study, the authors highlight how long-term, community-driven efforts can advance solutions to these problems. Chapter 2 highlights training gaps in urban medical training, including a lack of attention to cultural competence, medical mistrust, and the social determinants of health in standard curricula. Medical mistrust receives significant attention in this chapter, and it is rooted in historical injustices in American society—such as the Tuskegee Syphilis Study—to contemporary realities, such as racial bias in medical care. Chapter 2 discusses federal efforts to address the shortage of health professionals in urban areas, including the National Health Service Corps and Area Health Education Centres. Chapter 3 explores the relatively new concept of the social determinants of health (SDOH), defined as the conditions in which people are born, grow, live, work, and age that can affect their health outcomes individually and in society. Such as education, economic stability, environmental conditions, access to health services, and social support systems. The authors underline how urban infrastructure, such as housing and transportation policies and access to food, affects health. The case of the food desert is persuasively made, showing how limited access to affordable nutritious food can lead to chronic diseases such as diabetes and heart disease. The fourth chapter discusses primary care in urban environments. This chapter discusses barriers to accessing health services, such as high caseloads, few health care providers, and insurance restrictions. It also discusses the relationship between patients and healthcare providers, including how bias and systematic oppression impact the quality of healthcare that urban patients receive. The authors argue for a more patient-centred, holistic approach in which healthcare providers recognise their patients' life experiences and engage in collaborative efforts to remove barriers to healthcare. Chapter 5 explores the mental health gap in cities, while defining the difference between mental illness and mental health stress triggers. This chapter underlines the impact of community trauma, economic instability, and racial discrimination on mental health outcomes.

The chapters on trauma-based care are quite interesting, emphasizing the need to uncover the root causes of mental distress - systemic problems and not just symptoms. In the sixth chapter, they review tertiary patient management and discuss access to primary services in urban areas and how urban communities overuse emergency rooms. The authors discuss the challenges of discharging patients from hospitals, idealism, transportation issues, financial barriers, and the lack of care resources that facilitate hospitalization and long-term outcomes. They argue for an interdisciplinary approach that aligns nurses, doctors, social workers, and public health workers to improve care coordination.

The final chapter discusses the urban health gap at the time of COVID-19. They use Detroit as a case study to show how COVID-19 exacerbates existing health inequalities and results in COVID-19-related deaths among communities of colour occurring disproportionately. They call for policies and investments in urban health infrastructure. Urban Health: Practical Applications for Clinical-Based Learning has the greatest strength in its comprehen-sive interdisciplinary scope. Through innovative and engaging narratives, the authors seamlessly connect theory and practice, allowing readers to understand how urban health systems work.

Each chapter includes integrated case studies, examples, and reflective exercises to engage active learning, making this book an exceptional resource for medical students, nurses, and administrators in the health field. The family is also over-involved, leading to mistrust - another key aspect of strength based on community work and cultural competence. While many health textbooks take a paragraph to acknowledge racial differences, this book discusses the historical and structural reasons why trust may not be easy. This is very timely given the ongoing conversation about systemic racial bias in medicine and the need to foster patient trust by providing culturally inclusive care for all.

Of course, this book has limitations. Although urban health in the United States is comprehensively explored, the global perspective is less rich. International case studies would provide some more detailed working models for addressing urban health challenges, which exist all over the world, as food for thought in terms of best practices across health systems. In the long term, although this book does a good job of outlining barriers to care, I think it could also provide clearer, step-by-step advice to doctors on how to overcome the barriers it outlines. A greater focus on policy advocacy and systemic response would make this book more action-oriented.

Urban health is not adequately covered or disseminated in medical education data. In response to this gap, this book provides a clear structure for understanding urban health. It highlights patient-centred care, the social determinants of health, and interdisciplinary collaboration - all of which are key aspects of modern clinical practice. This is particularly relevant for students studying public health, nursing, and medicine; students at this university level are encouraged to think critically and apply their understanding to real-world problems. The case studies are based on real-life clinical experiences that reflect practical contexts to easily connect theoretical learning with practice. In real clinical practice environments, doctors regularly see patients with challenging social and economic difficulties. This book gives them the tools to help overcome these challenges and become a more compassionate and solution-oriented version of themselves in patient care.

*Urban Health: A Practice-Based Approach to Clinical Education* is an essential requirement for all those involved in urban healthcare. This book provides a nuanced and evidence-based approach to closing this gap and improving outcomes, relationships and systems, which is directly in line with the purpose of this site: to share quality information and content from the most credible sources. Although their own institutions are not yet world-class enough, there is still much room for improvement, especially through the expansion of international education and the provision of a more prescriptive policy agenda. This book is, nevertheless, an important contribution to medical education. The book encourages healthcare professionals to reconsider urban health, making it a valuable resource for those committed to improving health equity.

